# Evaluation of High-Throughput Genomic Assays for the Fc Gamma Receptor Locus

**DOI:** 10.1371/journal.pone.0142379

**Published:** 2015-11-06

**Authors:** Chantal E. Hargreaves, Chisako Iriyama, Matthew J. J. Rose-Zerilli, Sietse Q. Nagelkerke, Khiyam Hussain, Rosalind Ganderton, Charlotte Lee, Lee R. Machado, Edward J. Hollox, Helen Parker, Kate V. Latham, Taco W. Kuijpers, Kathleen N. Potter, Sarah E. Coupland, Andrew Davies, Michael Stackpole, Melanie Oates, Andrew R. Pettitt, Martin J. Glennie, Mark S. Cragg, Jonathan C. Strefford

**Affiliations:** 1 Cancer Sciences, Faculty of Medicine, University of Southampton, Southampton, SO16 6YD, United Kingdom; 2 Department of Hematology and Oncology, Nagoya University Graduate School of Medicine, Nagoya, Japan; 3 Department of Blood Cell Research, Sanquin Research and Landsteiner Laboratory, Academic Medical Center, University of Amsterdam, 1066 CX, Amsterdam, The Netherlands; 4 Molecular Pathology, University Hospital Southampton NHS Foundation Trust, Southampton, SO16 6YD, United Kingdom; 5 Department of Genetics, University of Leicester, Leicester, LE1 7RH, United Kingdom; 6 School of Health, University of Northampton, Northampton, NN2 7AL, United Kingdom; 7 Department of Pediatric Hematology, Immunology and Infectious Disease, Emma Children’s Hospital, Academic Medical Center, University of Amsterdam, 1105 AZ, Amsterdam, The Netherlands; 8 Department of Molecular and Clinical Cancer Medicine, Institute of Translational Medicine, University of Liverpool, Liverpool, L69 3GA, United Kingdom; INSERM, FRANCE

## Abstract

Cancer immunotherapy has been revolutionised by the use monoclonal antibodies (mAb) that function through their interaction with Fc gamma receptors (FcγRs). The low-affinity FcγR genes are highly homologous, map to a complex locus at 1p23 and harbour single nucleotide polymorphisms (SNPs) and copy number variation (CNV) that can impact on receptor function and response to therapeutic mAbs. This complexity can hinder accurate characterisation of the locus. We therefore evaluated and optimised a suite of assays for the genomic analysis of the FcγR locus amenable to peripheral blood mononuclear cells and formalin-fixed paraffin-embedded (FFPE) material that can be employed in a high-throughput manner. Assessment of TaqMan genotyping for *FCGR2A*-131H/R, *FCGR3A*-158F/V and *FCGR2B*-232I/T SNPs demonstrated the need for additional methods to discriminate genotypes for the *FCGR3A*-158F/V and *FCGR2B*-232I/T SNPs due to sequence homology and CNV in the region. A multiplex ligation-dependent probe amplification assay provided high quality SNP and CNV data in PBMC cases, but there was greater data variability in FFPE material in a manner that was predicted by the BIOMED-2 multiplex PCR protocol. In conclusion, we have evaluated a suite of assays for the genomic analysis of the FcγR locus that are scalable for application in large clinical trials of mAb therapy. These assays will ultimately help establish the importance of FcγR genetics in predicting response to antibody therapeutics.

## Introduction

Therapeutic monoclonal antibodies (mAbs) are effective against a range of human diseases from cancer to autoimmunity [1]. The majority of mAbs are of the IgG subclass and elicit a functional response through interaction with a family of Fcγ receptors (FcγR) expressed predominantly on myeloid and lymphoid cells [1].

The FcγR family consists of the high affinity receptor, FcγRI, that has been proposed to be fully occupied by circulating monomeric IgG, and the low affinity receptors, that are thought to engage with either circulating antibody:antigen immune-complexes or opsonised target cells, such as after therapeutic mAb administration [1]. The low affinity receptors are encoded by the *FCGR2A*, *FCGR2B*, *FCGR2C*, *FCGR3A* and *FCGR3B* genes, positioned within a highly homologous 200 kb locus on chromosome 1q23-24, that is subject to numerous single nucleotide polymorphisms (SNPs) and copy number variation (CNV) [2–6].

SNPs in the activating FcγR genes, *FCGR2A* (R131H) and *FCGR3A* (F158V), increase receptor affinity for IgG, and are associated with overall and progression-free survival in patients treated with mAb including rituximab [7, 8], cetuximab [9] and trastuzumab [10]. FcγRIIb expression can impair immunotherapy efficacy *in vivo* by suppressing activating FcγR signalling [11, 12] whilst the *FCGR2B*-232I/T SNP in the transmembrane domain of FcγRIIb impairs its inhibitory function and has been implicated in predisposition to autoimmune disease [3]. In addition, copy number variation (CNV), a well-established source of inter-individual genetic variation [5, 13, 14] has also been reported to target several FcγR genes and can alter surface FcγR protein expression and cellular function [2].

To maximise the efficacy of mAb therapy it will be vital to establish the genomic anatomy of the FcγR locus and assess the impact of its SNPs and CNVs on therapeutic response (reviewed in [6]). To do so, accurate assay systems are required for application to clinical trial material in a high-throughput manner, and that also have utility for the analysis of DNA extracted from formalin-fixed paraffin embedded (FFPE) tissues. These approaches will permit accurate clinical correlations to be obtained, with the potential of biomarker development to improve patient care.

Therefore, the aim of our study was to validate existing and novel allelic discrimination assays and further refine methods for deciphering CNV at the FcγR locus. Our study demonstrates that allelic-discrimination assays alone are insufficient for accurate genotyping at the FcγR locus and additional methods are required. Whilst MLPA is an attractive method for multiplex analysis of the locus, DNA quality is important and care should be taken when interpreting data from FFPE material [15].

## Methods

### Human samples and assessment of gDNA quality

Genomic DNA (gDNA) was extracted from peripheral blood mononuclear cells (PBMC) and FFPE material from three patient cohorts using the DNeasy Blood and Tissue Kit (Qiagen, GmbH, Hilden, Germany) and the QIAamp DNA FFPE Tissue Kit (Qiagen), respectively. Cohort 1 consisted of 2389 gDNA samples from a large, multi-centre PBL cohort. Cohort 2 contained matched PBMC and FFPE material from 11 indolent follicular lymphoma patient samples. Cohort 3 consisted of PBMC samples from 164 normal donors entering local blood transfusion services. All patients gave written consent to these studies according to the Declaration of Helsinki. This study was approved by the University of Southampton Faculty of Medicine Ethics Committee and the National Research Ethics Service Committee South Central—Hampshire.

A BIOMED-2 multiplex PCR assay was performed to assess the quality of gDNA obtained from FFPE material from cohort 2 as previously described [16]. In brief, samples were assessed for the amplification of PCR products of known size, 100–600 bp, with higher quality samples amplifying larger PCR fragments.

### TaqMan genotyping assays

The *FCGR2A*-131H/R (rs1801274), *FCGR3A*-158F/V (rs396991) and *FCGR2B*-232I/T (rs1050501) SNPs were genotyped in cohorts 1, 2 and 3 using commercially available (C_9077561_20 and C_25815666_10) and custom-designed TaqMan discrimination assays (Life Technologies, Paisley, UK), according to manufacturer’s instructions.

#### Multiplex ligation-dependent probe amplification

CNVs targeting the low-affinity FcγR genes were identified using an FcγR-specific MLPA assay (SALSA MLPA P110 and P111 probe mixes; MRC-Holland, Amsterdam, The Netherlands) according to the manufacturer’s instructions, as described previously [2, 17]. In brief, 100 or 200 ng of DNA for PBMC and FFPE material, respectively, was analysed in triplicate for CNVs across the locus from *FCGR2A* to *FCGR2B* and known SNPs in *FCGR2A* 131A/H, *FCGR3A* 158F/V, *FCGR2B* 232I/T, *FCGR2C* (57X/Q, rs10917661), *FCGR3B* HNA1a and HNA1b isoforms and in the promoter regions of *FCGR2B* and *FCGR2C* (-386 G>C, rs3219018 and -120A/T, rs34701572) using the Genetic Analysis System CEQ 8800 capillary electrophoresis machine and GenomeLab software (Beckman Coulter, High Wycombe, UK).

Prior to analysis, intra-sample data normalisation was performed using the Coffalyser.NET software (MRC-Holland) by comparing the peak height generated by probes detecting the genes of interest, against the peak heights generated by probes targeting control genes of known normal copy number. Inter-sample normalisation was performed by pooling 96 European Collection of Cell Cultures (ECACC) Human Random Control panel 1 (Porton Down, Public Health England, UK) gDNA samples to act as a reference sample, against which test cases were compared. Normalised MLPA data was analysed using Microsoft Excel 2010, error bars represent standard deviation (SD). Verification of SNP data was performed by Sanger sequencing of PCR products using standard approaches and primers outlined in **S1 Protocols** and **S1 Table**.

#### Paralogue ratio test

CNV data was confirmed at the FCGR3 locus using a paralogue ratio test (PRT) and restriction-enzyme-digest variant ratio assay using conditions as described previously [18–20]. *FCGR3A* and *FCGR3B* copy number was determined using the arginine to stop change that distinguishes *FCGR3A*. Duplicate calls from the PRT were combined with the two independent assays measuring restriction enzyme digestion ratios using a maximum-likelihood approach, which calls the most likely integer copy number given the results from all assays. For example, from a 2:1 ratio of the *FCGR3A*:*FCGR3B* REDVR with a total diploid copy number of 3, we would infer a copy number of 2 for *FCGR3A* and 1 for *FCGR3B*. HNA1a and HNA1b variants in *FCGR3B* were distinguished using the rs527909462 synonymous change.

#### Long-range PCR assay of *FCGR2B* and *FCGR2C*

In order to identify potential SNPs in the *FCGR2B* 232I/T TaqMan and sequencing primer binding sites, a long-range PCR assay to specifically amplify the *FCGR2B* and *FCGR2C* genes was adapted from [21, 22] with an extended annealing time of 12 minutes. In brief, 15 kb fragments were amplified using the Expand Long Template PCR System (Roche Applied Science) as described in the S1 Protocols, and analysed with Sanger Sequencing. The resulting PCR products were subsequently verified as described by Blank *et al* [23] for a unique 31 bp sequence found in intron six of *FCGR2B* but not *FCGR2C*.

## Results

### Accurate genotyping requires TaqMan analysis with Sanger validation

**Fig 1:**
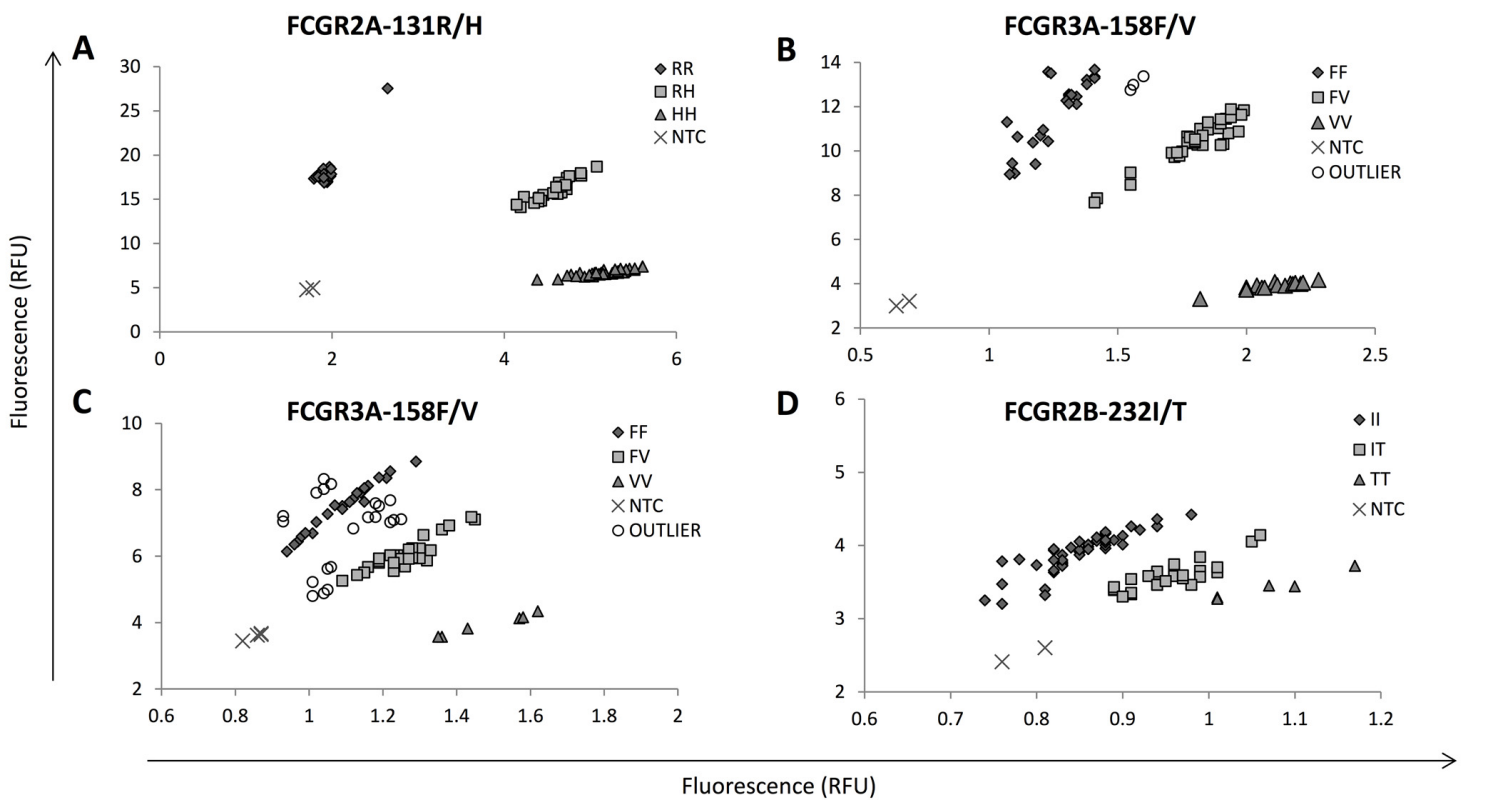
TaqMan allelic discrimination of *FCGR2A*-131H/R, *FCGR3A*-158F/V and *FCGR2B*-232I/T. (A) A representative *FCGR2A*-131R/H rs1801274 allelic discrimination plot, (B) a representative *FCGR3A*-158F/V rs396991 allelic discrimination plot with clear clustering of genotype calls, (C) a representative *FCGR3A*-158F/V rs396991 allelic discrimination plot where it is difficult to accurately distinguish between FF and FV genotype calls, (D) a representative custom TaqMan allelic discrimination plot of *FCGR2B*-232I/T rs1050501. All plots feature n = 24 samples performed in triplicate with non-template controls (NTCs). Axes represent relative fluorescence units (RFU). An outlier sample is defined as one where it was not possible to accurately genotype using Rotor-Gene Q software.

We first assessed commercial and bespoke TaqMan allelic discrimination assays in cohort 1 samples and showed that 1) the *FCGR2A*-131H/R assay performed consistently with clear discrimination of each genotype call (**Fig 1A**), was concordant with Sanger sequencing for the 10% of cases tested and showed allele frequencies of 19.9% RR, 46.3% RH and 34.6% HH (**Table 1**). 2) Whilst the *FCGR3A*-158 VV genotype clustered well, the FF and FV genotyping calls often clustered together (**Fig 1B and 1C**) in 315/2389 (13%) cases, and Sanger sequencing was required to accurately delineate these genotypes (**Table 1**). 3) The bespoke *FCGR2B*-232I/T TaqMan assay (**Fig 1D**) clearly and consistently identified II genotype calls, but Sanger sequencing was required to delineate the n = 662 non-II genotypes.

**Table 1:** FCGR genotype frequencies in cohort 1.

*Gene*	*SNP*	*Frequency n (%)*	*Chi-squared*	*P-value*
***FCGR2A***	RR	466 (19.76)	0.22	0.64
	RH	1102 (46.73)		
	HH	788 (33.42)		
	NVR*	2 (0.08)		
***FCGR3A***	FF	1095 (46.44)	0.08	0.8
	FV	1000 (42.41)		
	VV	260 (10.84)		
	NVR*	3 (0.13)		
***FCGR2BCustom TaqMan assay only***	II	1702 (72.18)	0.0002	0.99
	IT	525 (22.26)		
	TT	41 (1.74)		
	NVR*	90 (3.82)		
***FCGR2BCombined custom TaqMan and sequencing assays***	II	1710 (72.52)	2.82	0.09
	IT	531 (22.52)		
	TT	115 (4.88)		
	NVR*	2 (0.08)		

*NVR; no valid result

Chi-squared value <3.5 and a p-value of >0.05 were considered within Hardy-Weinberg equilibrium.

**Fig 2:**
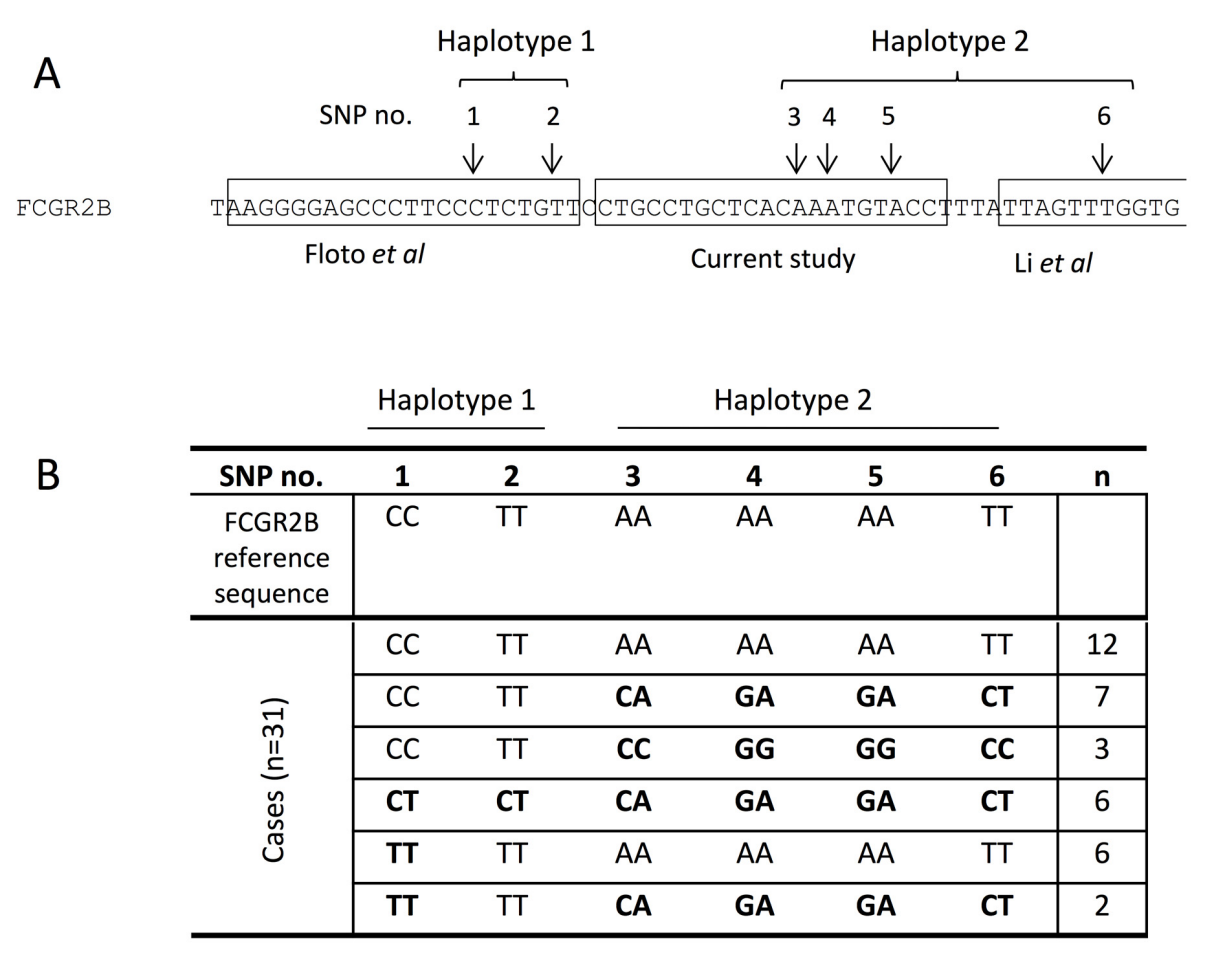
Polymorphisms in primer binding sites in intron 4 of *FCGR2B*. (A) We identified six polymorphisms in *FCGR2B* sequence used by primers for gene-specific sequencing of *FCGR2B*-232 SNP (boxed sequences) that form two haplotypes. (B) The genotype found at each polymorphic position (1–6) in the *FCGR2B* reference sequence and in the n = 31 cases tested is shown. Genotypes that differ from the reference are highlighted in bold. The two haplotypes formed six combinations of genotypes and the numbers of samples in each genotype group are indicated.

Whilst this data demonstrated that Taqman and Sanger validation are often both required for accurate genotyping, we did identify a problem with the *FCGR2B*-232I/T SNP, where gene-specific Sanger sequencing showed that 78/662 (11.8% of Sanger validated cases; 3.3% of the total cohort) were discordant with the TaqMan data. The most common conflict being samples genotyped by TaqMan as IT and TT by sequencing (n = 64/78). This disparity impacted on the prevalence of the TT genotype, whose frequency was 4.9% or 1.8% when the Sanger sequencing results were accepted or rejected, respectively (**Table 1**), the former being significantly higher than expected based on published frequencies of the TT genotype. To identify which assay was most accurate, we determined if primer binding site SNPs could explain this discordance. We applied gene-specific long-range PCR (LR-PCR) assays [21, 22] to amplify the individual *FCGR2B* and *FCGR2C* genes in n = 32 concordant or discordant cases for the TT genotype and used products for Sanger sequencing and allele-specific custom TaqMan analysis. As the discordancy remained, suggesting that the high level of sequence homology between *FCGR2B* and *FCGR2C* did not appear to confound our allelic discrimination, we searched for any unexpected sequence variants in the primer and probe binding sites using an additional PCR assay with a forward primer upstream of the sequencing primer binding sites. When applied to the *FCGR2B* gene template product, there was no variation in the TaqMan primer and probe binding sites but SNPs were present in the sequencing primer binding sites for the Floto *et al* [24], Li *et al* [25] and our gene-specific genotyping primers (**Fig 2**). This suggests that SNP variation in the Sanger primer binding sites may explain a proportion of the observed discordance at the *FCGR2B* locus (**S2 Table**). It was not possible to examine this effect at the *FCGR2C* locus, as we could not confirm a gene-specific LR-PCR product.

### Successful genotyping of FFPE material is dependent on DNA quality

The process of FFPE fixation can cause DNA fragmentation and cross-linking [26], compromising downstream molecular analysis, which can be particularly important for paralogous gene loci such as the FcγR region where longer amplification products may be required for inclusion of unique DNA sequence. Therefore, we aimed to determine the accuracy of Taqman assays in DNA obtained from matched PBMCs and FFPE material in 11 cases (cohort 2).

**Fig 3:**
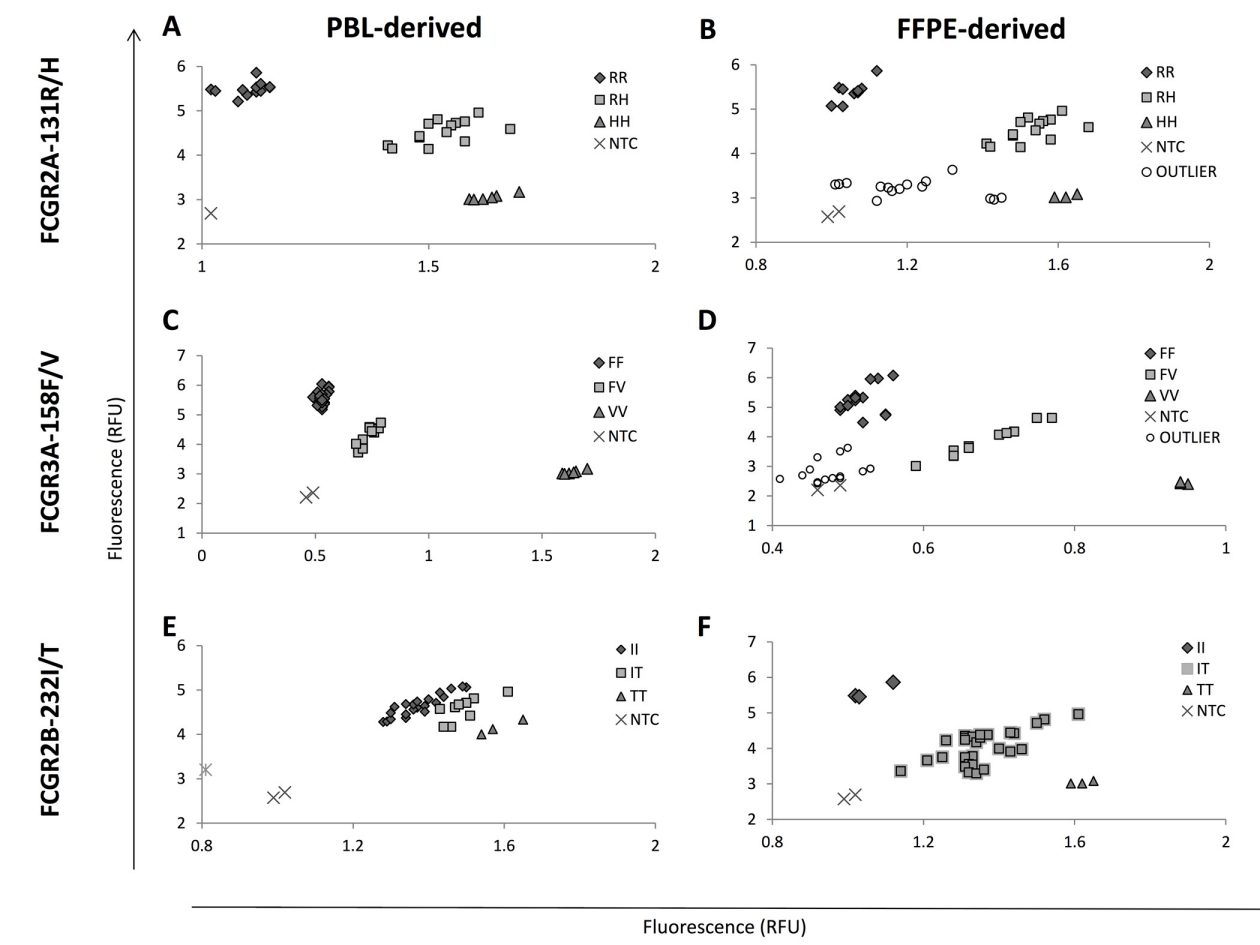
TaqMan allelic discrimination of FcγR SNPs in matched PBMC- and FFPE-derived material. TaqMan allelic discrimination assays were performed and compared in matched DNA from PBMC- (A, C, E) and FFPE- (B, D, F) derived material in n = 9 cases. Assays were performed in triplicate for SNPs (A-B) *FCGR2A*-131H/R rs1801274, (C-D) *FCGR3A*-158F/V rs396991 and (E-F) *FCGR2B*-232I/T rs1050501. All samples were performed in triplicate with non-template controls (NTCs). Axes represent relative fluorescence units (RFU). An outlier sample is defined as one where it was not possible to accurately genotype using Rotor-Gene Q software.

In an attempt to identify a method of stratified FFPE samples into those most likely to be processed successfully, we defined three groups based on the amplification of PCR fragments of known size (100–600 bp), using the BIOMED-2 primers; those that a) failed to amplify any product [n = 4], b) only amplified the 100 bp product [n = 3], or c) amplified a 200 bp product [n = 4] (**S3 Table**). Whilst, accurate genotyping data was obtained from all PBMC-derived samples (**Fig 3A, 3C and 3E**), 5/11 FFPE-derived samples failed genotyping analysis (4 with no BIOMED-2 amplification, and 1 with only the 100 bp product weakly visible by agarose gel electrophoresis), suggesting that successful FcγR genotyping is dependent on DNA quality and can be predicted using the BIOMED-2 primers.

### Multiplexed discrimination of FcγR CNV and SNPs in gDNA from PBMCs

**Fig 4:**
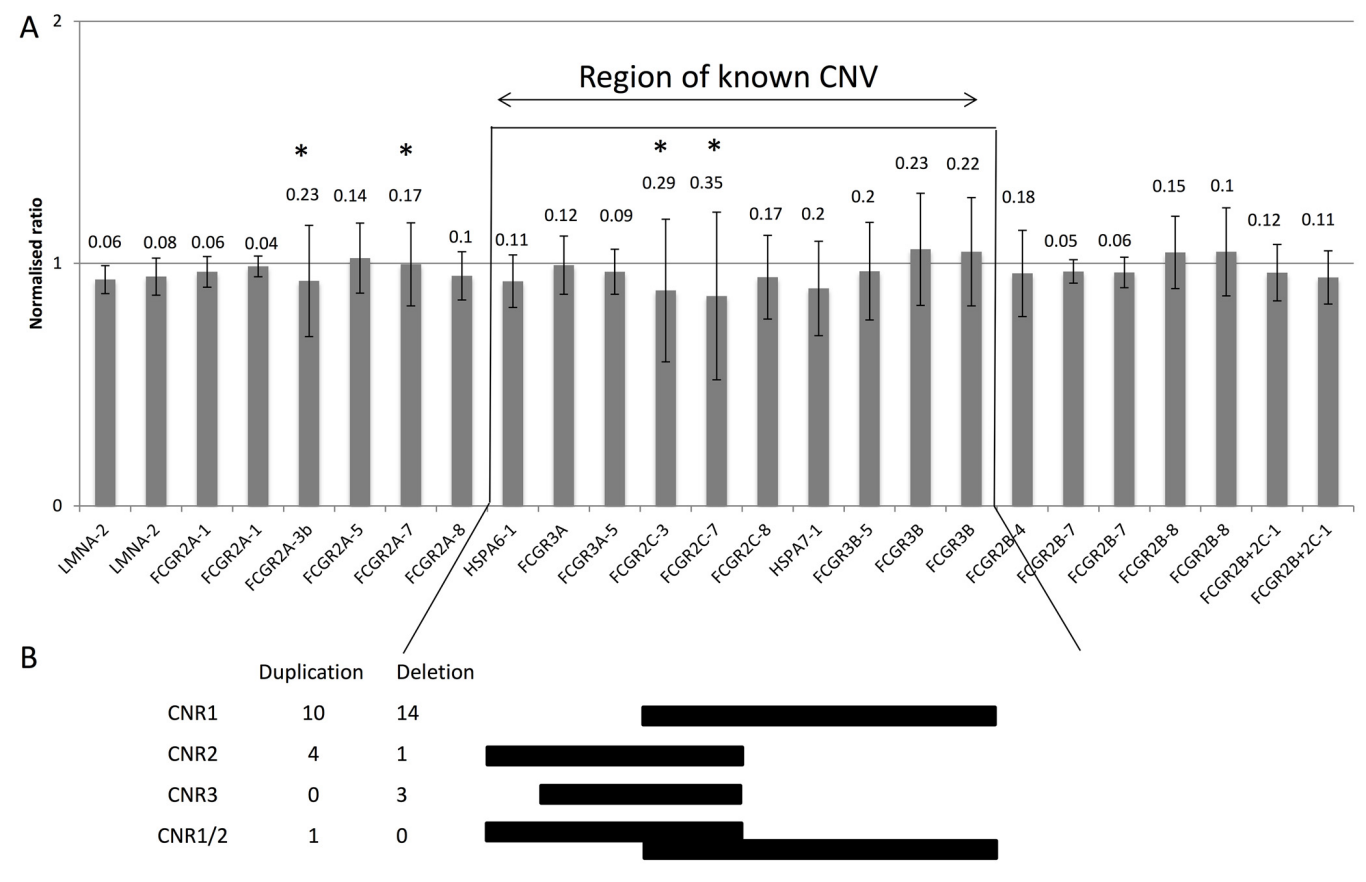
Performance of FcγR -targeted probes in healthy donors. (A) Probe binding performance was assessed by measuring the mean and SD of individual probes across our cohort of 164. Probes are represented in locus order. A normalised peak height ratio of 1 represents a diploid copy number. Error bars represent the mean +/- SD and the SD is shown above each probe. (B) Copy number regions (CNRs) 1 and 2 in healthy donors with observed numbers of duplication and deletion events. One donor showed CNV likely to include two duplications; one of the distal part containing *FCGR2C* and *FCGR3B* (CNR1) on one chromosome and one of the proximal part containing *FCGR3A* and *FCGR2C* (CNR2) on the other chromosome. * Probes in which the variability is a result of known genomic SNPs targeted by the probe.

In addition to the biological and clinical impact of FcγR SNPs, it is clear that CNV at the locus is also likely to have an important impact. Therefore, we investigated CNV and SNPs in combination using MLPA in cohort 3 cases (n = 164). Firstly, we assessed the assay’s performance by investigating the reproducibility of control CNV probes and FcγR gene-targeting probes by comparing the SD of the mean peak heights for each probe across the cohort, with an SD of ≤0.1 showing robust probe binding. In the case of control reference probes, an SD of less than 0.1 was observed in all probes, except probe 05–070.988325 (targets chromosome 5; SD = 0.11), and two probes on the X chromosome (X-110.253034 and X-110.253034) (**S1 Fig**), the latter of which is expected in a mixed population (96% male). For the FcγR gene probes, we observed increased binding variability at the *FCGR2C* and *FCGR3B* loci (SD = 0.2–0.36), due to CNV (**Fig 4A**). Variation was observed in *FCGR2C* exon 3 and exon 7 due to ORF status and presence of a splice site SNP, rs76277413, at the border of exon 7 and intron 7 [27], respectively. In addition, we also observed variation in binding of the *FCGR2A-3b* probe due to the presence of SNPs, rs9427398 and rs9427394, and the *FCGR2A*-7 probe due to rs382627.

Secondly, we analysed each case for the presence of CNV and observed copy number regions (CNRs) 1 and 2 as described by Niederer *et al* [4], affecting *FCGR3A* (9/164, 5.5%), *FCGR2C* (37/164, 22.6%) and *FCGR3B (*29/164, 17.7%), but not *FCGR2A* or *FCGR2B* (**Fig 4B**) [2, 22]. We did not observe either CNR 3, likely because this CNR is reported to be rare in Caucasians and more prevalent in those of East Asian descent [4] or the rare CNV event in *FCGR3A* alone.

Thirdly, SNP frequencies analysed by the MLPA assay were in Hardy-Weinberg equilibrium for *FCGR2A*-131R/H, *FCGR3A*-158F/V, *FCGR2B-*232I/T and *FCGR2C-*57X/Q in normal copy number individuals as previously reported [2].

Finally, PRT was used to confirm our MLPA data for *FCGR3A* and *FCGR3B* copy number, as well as *FCGR3B* HNA status [n = 56 cases], showing 100, 92.1 and 92.1% concordance, respectively, with all 5 discordant cases exhibiting a *FCGR3B* copy number state of two and three with PRT and MLPA, respectively (**S4 Table**). Multiple additional rounds of confirmatory MLPA and PRT analysis of these cases did not provide any concordance, including after repeat testing by PRT on sheared and un-sheared genomic DNA, which has been suggested to affect certain PRT assays [28]. This data may suggest that there are rare cases with a genomic architecture that confounds these PRT and/or MLPA approaches.

### Multiplex analysis of FcγR SNPs and CNV in gDNA from FFPE material

**Fig 5:**
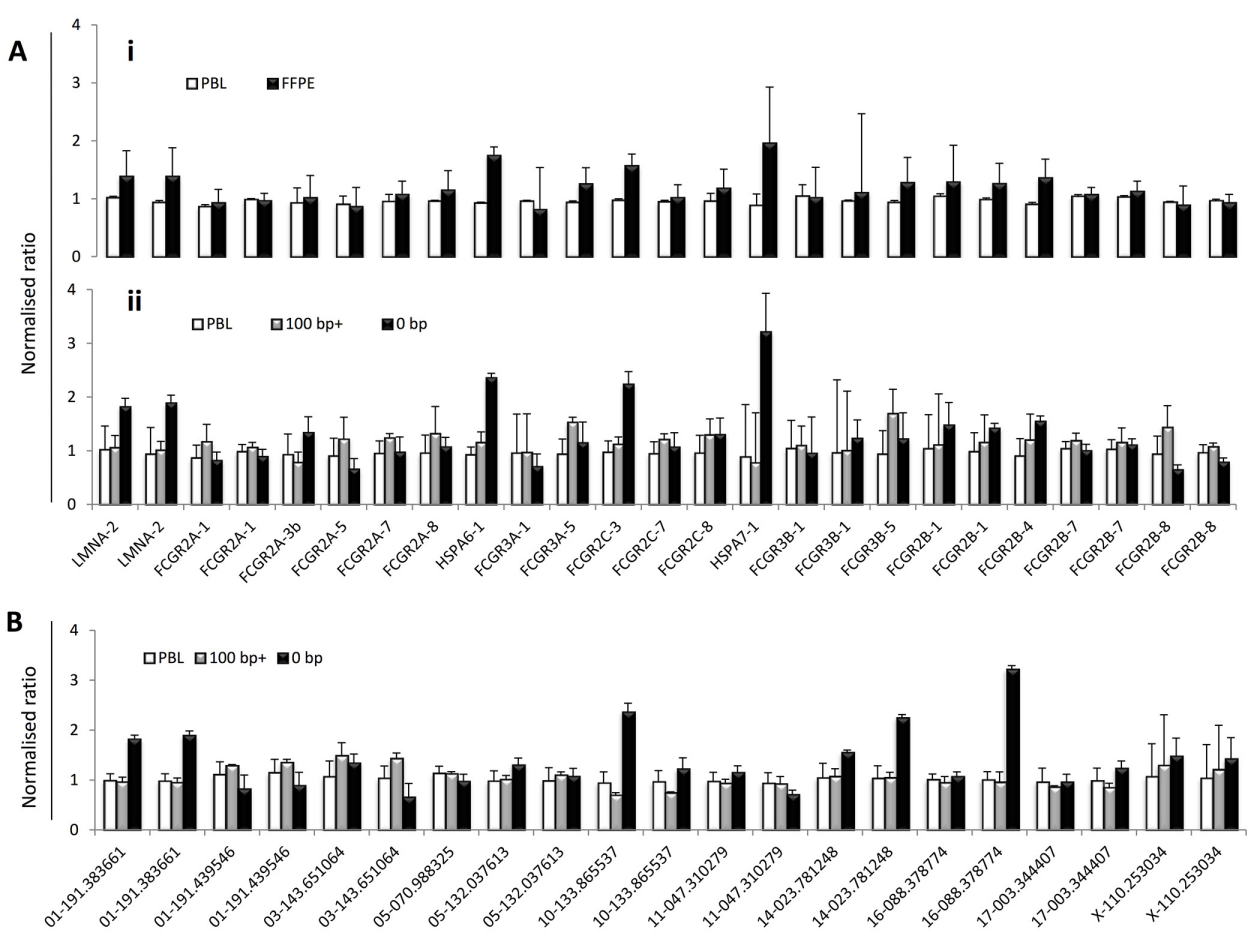
MLPA probe performance in gDNA from matched PBMC and FFPE-derived material. To evaluate the effects of FFPE treatment on MLPA performance, the variability of FcγR-specific probes was compared in (A) matched DNA from PBMCs and FFPE material. (B) The quality of FFPE material and its effect on FCGR probe MLPA performance was assessed by stratification of FFPE samples according to BIOMED-2 PCR fragment results. (C) The effects of the FFPE treatment process on the MLPA control probes were also assessed. Probes are represented in locus order with exons in brackets. A normalised peak height ratio of 1 represents a diploid copy number. Error bars represent mean +/- SD.

As an integrated SNP/CNV assay for the FcγR locus would likely need to be applicable to FFPE-extracted DNA, we assessed the success of MLPA to analyse the FcγR locus in lymphoma cases with matched DNA from PBMCs and FFPE (cohort 2, n = 7). Whilst probe performance in the PBMC samples was high, increased variability of probe binding was observed from matched FFPE material (**Fig 5A i**) with SD values ranging from 0.13–1.36 (**S6 Table**), which was most striking for probes targeting *HSPA6*, *FCGR2C* exon 3 and *HSPA7* (**S3 Table**). Mean probe binding of control and FcγR gene-targeting probes in FFPE material was stratified based on the BIOMED-2 PCR fragments amplified (n = 3 samples did not amplify any fragments, n = 4 amplified the 100–200 bp fragments) and compared to the mean probe binding in PBMC samples, showing that the most variability was observed in cases that failed to amplify any BIOMED-2 fragments (**Fig 5A ii and 5B**).

## Discussion

The purpose of the current study was to design optimal assays and guidelines for the investigation of the low affinity FcγR locus for use in clinical trials of mAb therapeutics using DNA samples obtained from PBMCs as well as from more challenging FFPE material. In doing so, our data supports an existing TaqMan allelic discrimination assay for *FCGR2A*-131H/R for the production of high-throughput reproducible, robust and high-quality data. However, we showed inconsistent genotype calling of the *FCGR3A*-158 FF and FV genotypes, probably due to the sequence homology between *FCGR3A* and *FCGR3B*, as the V allele sequence is present in both genes and both genes vary in copy number [2, 5]. We propose that accurate delineation of *FCGR3A* genotypes requires TaqMan combined with gene-specific Sanger sequencing.

Our custom *FCGR2B*-232I/T SNP TaqMan allelic discrimination assay robustly identified the II genotypes, but could not clearly delineate between IT and TT, likely due to sequence homology at the *FCGR2B* and *FCGR2C* loci. Sanger sequencing analysis of the non-II cases, using established *FCGR2B*-specific primers [24], identified a TT frequency of 4.88%, which is almost not within Hardy-Weinberg equilibrium, unlike our Taqman alone data (1.74%). The resultant discordancy between the two assay systems, that was rare in our cohort (3%), was investigated using a validated *FCGR2B* gene-specific LR-PCR assay that showed no evidence of any interference from other homologous genes. However, when the Taqman and Sanger primer and probe binding sites were sequenced, no variation was found in the TaqMan sequences, whilst SNP variation was identified in all the primers binding sites from our, and other published sequencing assays [24, 25]. The majority of the genotyping discordancy identified herein is probably due to this SNP variation, an observation that stresses the difficulty in primer design in the *FCGR2B* region, as the gene harbours SNP variation at sufficient frequency to affect primer binding and subsequent assay accuracy. For this reason TaqMan alone should be used for *FCGR2B*-232 genotyping, at least until additional more accurate assays can be validated. This observation has important clinical implications particularly if genotypes in this single inhibitory FcγR are ultimately used to stratify patients and guide treatment decisions.

Thousands of patient samples are stored in the form of FFPE material, representing an invaluable resource for clinical research if assays like those described here can overcome the issues of DNA quality and fragmentation inherent to FFPE. In our study, FFPE DNA that did not amplify at least the 100 bp BIOMED-2 PCR product consistently failed genotyping, suggesting stratification of FFPE material by DNA quality should precede FcγR genotyping to maximise success and assure data quality.

Our multiplex analysis of SNPs and CNV regions using MLPA was highly effective for the analysis of PBMC cases producing reproducible and high-quality data. Our CNV data confirmed previously published data [2], and also identified one individual who showed two duplications; one of the proximal part containing *FCGR3A* and *FCGR2C*, and one of the distal part containing *FCGR2C* and *FCGR3B*. These two duplications in one individual are likely to be present on different chromosomes, although the possibility that they are located next to each other on one chromosome cannot be ruled out.

MLPA data was concordant with our PRT results in the majority of cases (91%), with only a fraction of samples (9%) showing discordancy for *FCGR3B* copy number and HNA SNP isoforms. For these discordant cases, the *FCGR3B* copy number duplication observed by MLPA was also accompanied by duplication of the HNA SNP isoform probes and gain of *FCGR2C* CNV and *FCGR2C*-57X/Q SNP probes, an observation that has previously been linked to increased *FCGR3B* copy number [2, 4, 20]. On the other hand, the PRT copy number results were further supported by restriction enzyme digest ratios leading to a confident assignment of *FCGR3B* copy number by maximum likelihood. This leads to a question regarding the true copy number of this region in these cases. The discordancy is likely due to the structure of the *FCGR3B* locus that remains to be elucidated at the genomic level, as both assays performed with high reproducibility using technical replicates and generated identical results upon sample re-testing. Further analysis will be required to dissect the genomic architecture of this region.

Finally, we show that FFPE-derived samples should be stratified by applying the BIOMED-2 multiplex PCR protocol to identify cases most likely to be successfully analysed. The increased probe binding variability observed in the FFPE material being a result of somatically acquired FcγR CNV cannot be excluded. However we feel this is unlikely as the observed variability correlated with DNA quality assessed by the BIOMED-2 protocol.

mAbs have revolutionised cancer treatment and further large-scale international clinical trials of novel agents are expected. Existing literature has shown that FcγR SNPs can affect overall and progression-free survival following mAb administration [7–9], while FcγR CNV remains largely untested in this field. It is therefore essential that future studies investigating the contribution of FcγR genetics in immunotherapy apply sufficiently robust assays to ensure accurate and conclusive clinical associations. This study highlights the complexity of the FcγR locus and the difficulty in the design and interpretation of genetic assays. We make recommendations on the choice and analysis of assays depending on the quality of available material, information required and necessary validation experiments. Given the rapidly advancing nature of genomics and the implementation of next generation sequencing (NGS) technologies, the assays described here will be further required for validation of such NGS data or at least serve to highlight that care must be taken in analysing the homologous FcγR gene locus.

In conclusion, we have evaluated a suite of assays for the genomic analysis of the FcγR locus that are scalable for application in large clinical trials of antibody therapy. This work will ultimately provide a detailed architecture of the region and establish the importance of FcγR genetics in predicting response to antibody therapeutics.

## Supporting Information

S1 Fig Reproducibility of MLPA control gene-targeted probes in normal donors. Probe binding reproducibility was assessed by measuring the SD of individual probes across our cohort of normal donors. Error bars represent mean +/- SD. SD of probe binding is shown above the error bars.
(TIFF)

S1 Protocols (DOCX)

S1 Table Primer and probe sequences used in study. (DOCX)

S2 Table Frequencies of SNPs found in FCGR2B gene-specific sequencing primer binding sites. (DOCX)

S3 Table Comparison of quality characteristics of gDNA extracted from FFPE material. (DOCX)

S4 Table Comparison of MLPA and PRT data for FCGR3B copy number and HNA isoform genotypes. (DOCX)

S5 Table SNP frequencies observed by MLPA analysis in a cohort of normal donors. (DOCX)

S6 Table Mean and SD values for MLPA FCGR-targeting probes across matched PBL and FFPE sample preparations. (DOCX)

## Abbreviations

CNV: copy number variation
FFPE: formalin-fixed paraffin-embedded
FcγR: Fc gamma receptor
LR-PCR: long-range PCR
mAb: monoclonal antibody
MLPA: multiplex ligation-dependent probe amplification
PRT: paralogue ratio test
RFU: relative fluorescence units
SNP: single nucleotide polymorphism
