# Correction: Evaluation of High-Throughput Genomic Assays for the Fc Gamma Receptor Locus

**DOI:** 10.1371/journal.pone.0145040

**Published:** 2016-03-23

**Authors:** Chantal E. Hargreaves, Chisako Iriyama, Matthew J. J. Rose-Zerilli, Sietse Q. Nagelkerke, Khiyam Hussain, Rosalind Ganderton, Charlotte Lee, Lee R. Machado, Edward J. Hollox, Helen Parker, Kate V. Latham, Taco W. Kuijpers, Kathleen N. Potter, Sarah E. Coupland, Andrew Davies, Michael Stackpole, Melanie Oates, Andrew R. Pettitt, Martin J. Glennie, Mark S. Cragg, Jonathan C. Strefford

Fig 4 is incorrect. The figure is an older version, which contains an inaccurate summary of data. The authors have provided a corrected version here.

**Fig 4:**
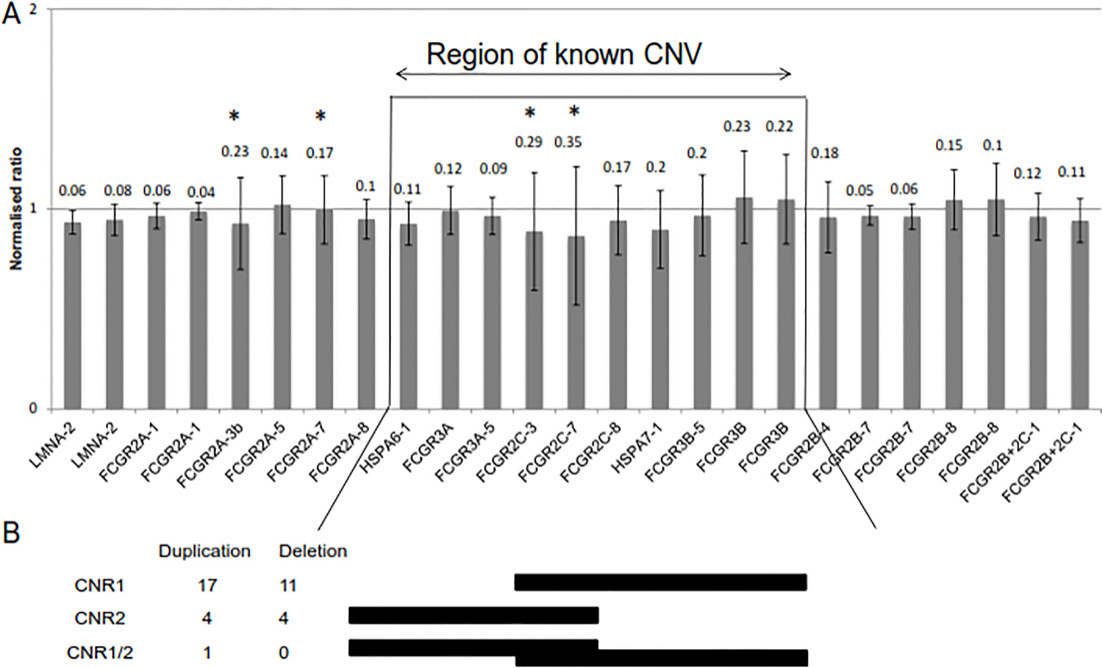
Performance of FcγR -targeted probes in healthy donors. (A) Probe binding performance was assessed by measuring the mean and SD of individual probes across our cohort of 164. Probes are represented in locus order. A normalised peak height ratio of 1 represents a diploid copy number. Error bars represent the mean +/- SD and the SD is shown above each probe. (B) Copy number regions (CNRs) 1 and 2 in healthy donors with observed numbers of duplication and deletion events. One donor showed CNV likely to include two duplications; one of the distal part containing *FCGR2C* and *FCGR3B* (CNR1) on one chromosome and one of the proximal part containing *FCGR3A* and *FCGR2C* (CNR2) on the other chromosome. * Probes in which the variability is a result of known genomic SNPs targeted by the probe.

## References

[1] Hargreaves CE, Iriyama C, Rose-Zerilli MJJ, Nagelkerke SQ, Hussain K, Ganderton R, et al (2015) Evaluation of High-Throughput Genomic Assays for the Fc Gamma Receptor Locus. PLoS ONE 10(11): e0142379 doi:10.1371/journal.pone.0142379 26545243 10.1371/journal.pone.0142379 PMC4636148

