# Supplementary figures and images for: Evaluation of High-Throughput Genomic Assays for the Fc Gamma Receptor Locus
Source: PLoS One. 2015 Nov 6;10(11):e0142379. doi: 10.1371/journal.pone.0142379 (PMC4636148; doi:10.1371/journal.pone.0142379)

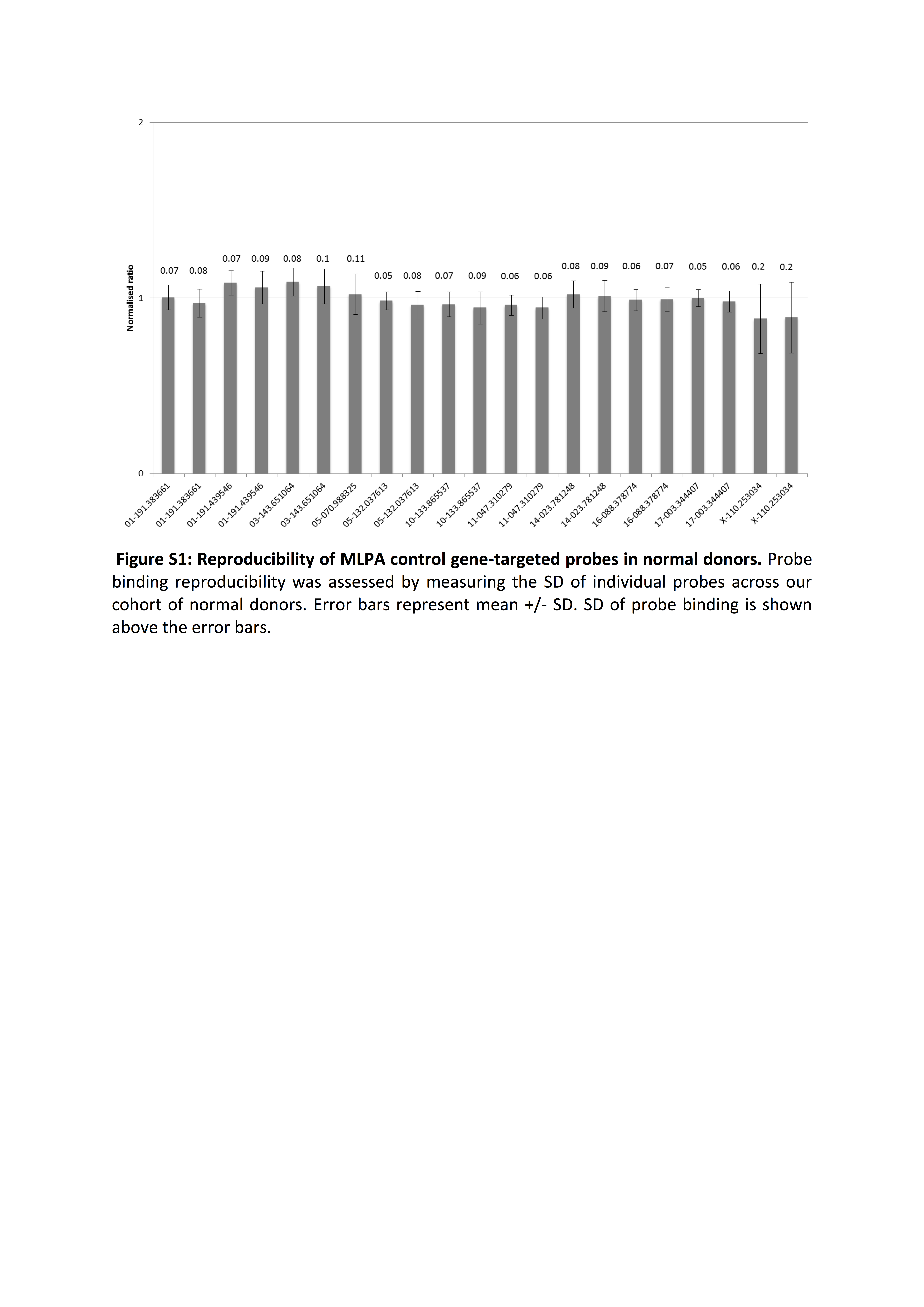

Supplement: S1 Fig — Probe binding reproducibility was assessed by measuring the SD of individual probes across our cohort of normal donors. Error bars represent mean +/- SD. SD of probe binding is shown above the error bars. (TIFF) [file pone.0142379.s001.tiff]
