# Supplementary material for: Evaluation of High-Throughput Genomic Assays for the Fc Gamma Receptor Locus
Source: PLoS One. 2015 Nov 6;10(11):e0142379. doi: 10.1371/journal.pone.0142379 (PMC4636148; doi:10.1371/journal.pone.0142379)
Supplement: S1 Protocols — (DOCX) [file pone.0142379.s002.docx]

# ****S1 protocols****

## gDNA extraction from FFPE material

gDNA was extracted from FFPE material by following the manufacturer’s instructions by heat-treating samples for 1 hr or until completely dissolved (FFPE) and further slices from the same samples were extracted again by heat-treating for a maximum time of 1 hr, regardless of whether the sample was fully dissolved or not (FFPE-alt).

## TaqMan genotyping assays

Samples were prepared in triplicate with genotyping controls containing sequence-confirmed SNPs (Coriell Cell Repository, Camden, NJ, USA) and non-template controls (NTCs). Each genotyping run was prepared using the CAS-1200 PCR setup robot (Corbett Life Science, Qiagen), and amplification and allelic discrimination was performed using a Corbett Rotor-Gene 6000 (Corbett Life Science) and Rotor-Gene Q series Software 2.0.2 (Build 4), respectively.

## Sanger sequencing

Genotype results from genotyping assays and MLPA were confirmed by direct sequencing of PCR products. Touchdown PCR cycling conditions were 95°C for 10 minutes, 8 cycles of 95°C for 30 seconds, 65°C for 45 seconds with a drop of 1°C per cycle until an annealing temperature of 57°C is reached, and 72°C for 1 minute 30 seconds, followed by 37 cycles of 95°C for 30 seconds, 57°C for 45 seconds, and 72°C for 1 minute 30 seconds. PCR products were purified by MinElute 96 UF PCR purification kit (Qiagen) or the ExoSAP method and SNPs were confirmed by Sanger sequencing (Source BioScience, Cambridge, UK). The primers used for PCR and Sanger sequencing are listed in Table 1.

## Long Range PCR

PCR cycling conditions were 94°C for 2 minutes, followed by 10 cycles of 94°C for 10 seconds, 60°C for 30 seconds and 68°C for 12 minutes, followed by 20 cycles of 94°C for 15 seconds, 60°C for 30 seconds and 68°C for 12 minutes, with an additional 20 seconds after each cycle at 68°C, followed by a final elongation at 72°C for 7 minutes. PCR products were purified using 1U/µL Shrimp Alkaline Phosphatase (New England Biolabs) and 20U/µL Exonuclease I and incubation at 37°C for 30 minutes followed by 95°C for 5 minutes.

## PRT

For the PRT, forward primer (5’-ATGATCTGGCCCTGAAACTC-3’) and either FAM or HEX labelled reverse primers (5’-TGAGTTCAAGAAAGCAGTTTGG-3’) were used. REDVR assays amplified two regions in duplex, using the primers 38L (5’FAM-AAGACTGAGCCACCAAGCAT-3’), 38R (5’-CTCCCTGGCACTTCAGAGTC-3’), 234L (5’-TTTTGCAGTGGACACAGGAC-3’), and 234R (5’HEX-GGGTTGCAAATCCAGAGAAA-3’). Fragments were analysed using an ABI 3130xl Genetic Analyzer (Applied Biosystems, Warrington, UK), with an injection time of 30 seconds. Peak areas were recorded for both FAM- and HEX-labelled products using GeneMapper software (Applied Biosystems) and mean ratios were used in conjunction with reference standards from the Human Random Control collection (Public Health England, Salisbury, UK) to calibrate each experiment.
