# Supplementary material for: Evaluation of High-Throughput Genomic Assays for the Fc Gamma Receptor Locus
Source: PLoS One. 2015 Nov 6;10(11):e0142379. doi: 10.1371/journal.pone.0142379 (PMC4636148; doi:10.1371/journal.pone.0142379)
Supplement: S1 Table — (DOCX) [file pone.0142379.s003.docx]

**S1 Table: Primer and probe sequences used in study.**

| **Gene/SNP** | **Primer** | **Primer sequence** |
| --- | --- | --- |
| **FCGR2A R131H** rs1801274 | Forward PCR primer | CATATATTGCCTATAAGAGAATGCT |
|  | Reverse PCR primer | CCTGACTACCTATTACCTGGGA |
| **FCGR3A F158V**  rs396991 | Forward PCR primer | CCTCTAATAGGGCAATTCATCATT |
|  | Reverse PCR and sequencing primer | AGATGTGGCTTCTGCTCCTG |
|  | Forward sequencing primer | TGCTCTGCATAAGGTCACATATT |
| **FCGR2B I232T**  rs1050501 | Floto *et al* Forward primer ^21^ | AAGGGGAGCCCTTCCCTCTGTT |
|  | Floto *et al* Reverse primer ^21^ | CATCACCCACCATGTCTCAC |
|  | custom TaqMan forward primer | GATGGGGATCATTGTGGCTGT |
|  | custom TaqMan reverse primer | AGGCCACTACAGCAGCAACAAT |
|  | Custom TaqMan 232I probe | ACTGGGATTGCTGTAGC |
|  | Custom TaqMan 232T probe | ACTGGGACTGCTGTAGC |
|  | Alternative FCGR2B-specific sequencing forward primer | ctgcctgctcacaaatgta |
|  | Alternative FCGR2B-specific sequencing reverse primer | cactgctctccccaagac |
| **FCGR2C X57Q**  rs10917661 | Forward PCR primer | TTTCGAGGGTGTTTCTCTTG |
|  | Reverse PCR primer | AAAGCACAGTCAGATGCACA |
| **FCGR2B/2C promoter SNPs**  rs3219018 and rs34701572 | Forward PCR primer | caggggaataatgaggatgA |
|  | Reverse PCR primer | tcagtggcaaggacaggta |
| **FCGR3B HNA isoforms**  rs200688856 and rs527909462 | Forward PCR primer | caatgcagcagcctatatctac |
|  | Reverse PCR primer | atggccataagatattgggaaa |
