# Supplementary material for: Evaluation of High-Throughput Genomic Assays for the Fc Gamma Receptor Locus
Source: PLoS One. 2015 Nov 6;10(11):e0142379. doi: 10.1371/journal.pone.0142379 (PMC4636148; doi:10.1371/journal.pone.0142379)
Supplement: S2 Table — (DOCX) [file pone.0142379.s004.docx]

## S2 Table: Frequencies of SNPs found in *FCGR2B* gene-specific sequencing primer binding sites.

| **SNPs** | **FCGR2B rs name** | **Variation** | **Primer affected** | **Haplotype block** | **Frequencies** | |
| --- | --- | --- | --- | --- | --- | --- |
| 1  2 | rs2793082  rs2793081 | C/T  C/T | Floto *et al*^21^ | 1 | CC | 64.5 |
|  |  |  |  |  | CT | 9.7 |
|  |  |  |  |  | TT | 25.8 |
| 3  4  5 | rs1674755  rs369224649  rs1674756 | A/C  A/G  A/G | Hargreaves *et al* | 2 | Germline | 48.4 |
|  |  |  |  |  | Heterozygous | 38.7 |
|  |  |  |  |  | Variant | 12.9 |
| 6 | rs1771554 | C/T | Li *et al*^22^ |  |  |  |
