# Supplementary material for: Evaluation of High-Throughput Genomic Assays for the Fc Gamma Receptor Locus
Source: PLoS One. 2015 Nov 6;10(11):e0142379. doi: 10.1371/journal.pone.0142379 (PMC4636148; doi:10.1371/journal.pone.0142379)
Supplement: S3 Table — (DOCX) [file pone.0142379.s005.docx]

**S3 Table: Comparison of quality characteristics of gDNA extracted from FFPE material.**

|  | **DNA concentration (ng/µL)** | **DNA absorbance (260/280)** | **BIOMED-2 result (bp)** |
| --- | --- | --- | --- |
| Sample 1 | 52.4 | 1.96 | 100 |
| Sample 2 | 530.3 | 1.98 | 100 |
| Sample 3 | 298.6 | 1.78 | 0 |
| Sample 4 | 238.9 | 1.81 | 0 |
| Sample 5 | 660.8 | 1.98 | 100 |
| Sample 6 | 211.4 | 1.82 | 0 |
| Sample 7 | 1240.6 | 1.98 | 200 |
