# Supplementary material for: Evaluation of High-Throughput Genomic Assays for the Fc Gamma Receptor Locus
Source: PLoS One. 2015 Nov 6;10(11):e0142379. doi: 10.1371/journal.pone.0142379 (PMC4636148; doi:10.1371/journal.pone.0142379)
Supplement: S4 Table — (DOCX) [file pone.0142379.s006.docx]

**S4 Table: Comparison of MLPA and PRT data for FCGR3B copy number and HNA isoform genotypes.**

| **Sample** | **MLPA data** | | **PRT data** | |
| --- | --- | --- | --- | --- |
|  | **FCGR3B copy number** | **FCBR3B HNA 1A/B** | **FCGR3B copy number** | **FCBR3B HNA 1A/B** |
| CRA 37 | 3 | abb | 2 | bb |
| CRA 41 | 3 | abb | 2 | bb |
| CRA 54 | 3 | aab | 2 | Ab |
| CRA 55 | 3 | aab | 2 | Ab |
| CRA 78 | 3 | aab | 2 | ab |
