# Supplementary material for: Evaluation of High-Throughput Genomic Assays for the Fc Gamma Receptor Locus
Source: PLoS One. 2015 Nov 6;10(11):e0142379. doi: 10.1371/journal.pone.0142379 (PMC4636148; doi:10.1371/journal.pone.0142379)
Supplement: S5 Table — (DOCX) [file pone.0142379.s007.docx]

## S5 Table: SNP frequencies observed by MLPA analysis in a cohort of normal donors.

| **Gene** | **Genotype** | **N** | **%** |
| --- | --- | --- | --- |
| **FCGR2A** | **RR** | 37 | 22.29 |
|  | **RH** | 92 | 55.42 |
|  | **HH** | 28 | 16.87 |
| **FCGR3A** | **FF** | 55 | 33.13 |
|  | **FV** | 80 | 48.19 |
|  | **VV** | 13 | 7.83 |
|  | **F** | 1 | 0.60 |
|  | **V** | 0 | 0.00 |
|  | **FFF** | 4 | 2.41 |
|  | **FFV** | 1 | 0.60 |
|  | **FVV** | 0 | 0.00 |
|  | **VVV** | 0 | 0.00 |
| **FCGR2B** | **II** | 119 | 74.38 |
|  | **IT** | 28 | 17.50 |
|  | **TT** | 0 | 0.00 |
| **FCGR2C** | **XX** | 88 | 54.32 |
|  | **XQ** | 24 | 14.81 |
|  | **QQ** | 3 | 1.85 |
|  | **X** | 9 | 5.56 |
|  | **Q** | 5 | 3.09 |
|  | **XXX** | 15 | 5.86 |
|  | **XXQ** | 0 | 0.00 |
|  | **XQQ** | 2 | 1.23 |
|  | **QQQ** | 2 | 1.23 |
| **FCGR3B** | **1A/1A** | 7 | 4.27 |
|  | **1A/1B** | 61 | 37.20 |
|  | **1B/1B** | 52 | 31.71 |
|  | **1A** | 3 | 1.83 |
|  | **1B** | 10 | 6.10 |
|  | **1A/1A/1A** | 0 | 0.00 |
|  | **1A/1A/1B** | 8 | 4.88 |
|  | **1A/1B/1B** | 8 | 4.88 |
|  | **1B/1B/1B** | 0 | 0.00 |

The frequencies of SNP genotypes were assessed by MLPA. Genotypes in normal copy number individuals were assessed for being within Hardy-Weinberg equilibrium using a Chi-squared value of <3.5 and a p-value of >0.05.
