# Supplementary material for: Evaluation of High-Throughput Genomic Assays for the Fc Gamma Receptor Locus
Source: PLoS One. 2015 Nov 6;10(11):e0142379. doi: 10.1371/journal.pone.0142379 (PMC4636148; doi:10.1371/journal.pone.0142379)
Supplement: S6 Table — (DOCX) [file pone.0142379.s008.docx]

**S6 Table: Mean and SD values for MLPA FCGR-targeting probes across matched PBL and FFPE sample preparations.**

|  | **PBL** | | **FFPE total** | | **FFPE 0bps** | | **FFPE 100bp+** | |
| --- | --- | --- | --- | --- | --- | --- | --- | --- |
|  | **Mean** | **SD** | **Mean** | **SD** | **Mean** | **SD** | **Mean** | **SD** |
| LMNA-2 | 1.02 | 0.02 | 1.39 | 0.44 | 1.82 | 0.22 | 1.07 | 0.15 |
| LMNA-2 | 0.94 | 0.02 | 1.39 | 0.49 | 1.89 | 0.16 | 1.01 | 0.14 |
| FCGR2A-1 | 0.87 | 0.03 | 0.93 | 0.23 | 0.82 | 0.32 | 1.01 | 0.15 |
| FCGR2A-1 | 0.99 | 0.01 | 0.97 | 0.13 | 0.89 | 0.08 | 1.02 | 0.13 |
| FCGR2A-3b | 0.93 | 0.25 | 1.02 | 0.38 | 1.34 | 0.19 | 0.78 | 0.29 |
| FCGR2A-5 | 0.90 | 0.18 | 0.86 | 0.33 | 0.66 | 0.40 | 1.02 | 0.19 |
| FCGR2A-6 | 0.96 | 0.02 | 1.08 | 0.22 | 0.98 | 0.07 | 1.16 | 0.28 |
| FCGR2C-7 | 0.96 | 0.01 | 1.18 | 0.33 | 1.30 | 0.50 | 1.09 | 0.17 |
| FCGR2A-7 | 0.96 | 0.02 | 1.15 | 0.14 | 1.07 | 0.19 | 1.21 | 0.08 |
| HSPA6-1 | 0.93 | 0.01 | 1.75 | 0.72 | 2.36 | 0.71 | 1.29 | 0.23 |
| FCGR3A-5 | 0.94 | 0.03 | 1.26 | 0.20 | 1.15 | 0.13 | 1.34 | 0.23 |
| FCGR3A | 0.96 | 0.02 | 0.82 | 0.22 | 0.71 | 0.10 | 0.90 | 0.26 |
| FCGR2B-4 | 0.90 | 0.01 | 1.36 | 0.33 | 1.55 | 0.30 | 1.22 | 0.30 |
| FCGR2C-4 | 0.98 | 0.03 | 1.57 | 0.97 | 2.24 | 0.93 | 1.06 | 0.72 |
| FCGR2C-6 | 0.95 | 0.02 | 1.02 | 0.52 | 1.07 | 0.36 | 0.99 | 0.67 |
| HSPA7-1 | 0.89 | 0.01 | 1.96 | 1.36 | 3.21 | 1.11 | 1.02 | 0.34 |
| FCGR3B-5 | 0.94 | 0.03 | 1.28 | 0.28 | 1.22 | 0.09 | 1.32 | 0.38 |
| FCGR3B | 1.05 | 0.03 | 1.02 | 0.43 | 0.96 | 0.45 | 1.07 | 0.48 |
| FCGR3B | 0.96 | 0.04 | 1.11 | 0.63 | 1.23 | 0.94 | 1.01 | 0.41 |
| FCGR2B-7 | 1.04 | 0.03 | 1.07 | 0.13 | 1.01 | 0.14 | 1.12 | 0.11 |
| FCGR2B-7 | 1.03 | 0.02 | 1.13 | 0.17 | 1.11 | 0.27 | 1.14 | 0.11 |
| FCGR2B-8 | 0.94 | 0.01 | 0.89 | 0.33 | 0.65 | 0.40 | 1.07 | 0.09 |
| FCGR2B-8 | 0.97 | 0.02 | 0.93 | 0.15 | 0.79 | 0.06 | 1.04 | 0.07 |
| FCGR2B-1 | 1.04 | 0.03 | 1.29 | 0.35 | 1.48 | 0.50 | 1.15 | 0.09 |
| FCGR2B-1 | 0.99 | 0.02 | 1.26 | 0.32 | 1.42 | 0.48 | 1.14 | 0.10 |
